# Hand-assisted laparoscopic surgery compared with open resection for mid and low rectal cancer: a case-matched study with long-term follow-up

**DOI:** 10.1186/s12957-015-0616-4

**Published:** 2015-06-10

**Authors:** Xile Zhou, Fanlong Liu, Caizhao Lin, Qihan You, Jinsong Yang, Wenbin Chen, Jiahe Xu, Jianjiang Lin, Xiangming Xu

**Affiliations:** Department of Colorectal Surgery, the First Affiliated Hospital, College of Medicine, Zhejiang University, 79 Qingchun Road, Hangzhou, Zhejiang 310003 China; Department of Pathology, the First Affiliated Hospital, College of Medicine, Zhejiang University, 79 Qingchun Road, Hangzhou, Zhejiang 310003 China; Department of Radiation Oncology, the First Affiliated Hospital, College of Medicine, Zhejiang University, 79 Qingchun Road, Hangzhou, Zhejiang 310003 China

**Keywords:** Rectal neoplasms, Hand-assisted Laparoscopic Surgery (HALS), Total mesorectal excision

## Abstract

**Background:**

This study was designed to compare the long-term surgical outcomes of patients with mid and low rectal cancer after open or hand-assisted laparoscopic surgery (HALS).

**Methods:**

A case-matched controlled prospective analysis of 116 patients who underwent hand-assisted laparoscopic surgery (HALS) for stage I to III mid and low rectal cancer from 2005 to 2010 was performed. Contemporary patients who underwent open rectal surgery were matched to the HALS group at the ratio of 1:1. The perioperative clinical outcomes, postoperative pathology, and survival outcomes were compared between the groups.

**Results:**

The patient characteristics between the two groups were comparable. Ninety patients in the open group and 85 in the HALS group received sphincter-preserving surgery. HALS resulted in less blood loss and wound infection, faster return to oral diet, shorter postoperative hospital stay, and longer operating time. The two groups had similar complication rates. Lymph node retrieval and involvement of circumferential and distal margins were similar for both procedures. Cumulative incidences of locoregional recurrence, disease-free, or overall survival rates were statistically similar.

**Conclusions:**

This study suggests that HALS for mid and low rectal cancer is acceptable in terms of short-term clinical outcomes and long-term survival results.

## Background

Recent findings have shown that laparoscopic-assisted proctectomy (LAP) has equivalent survival outcomes compared with open resection for mid and low rectal cancer [1–6]. However, LAP or robotic surgery [7] is limited by the technical difficulties and long steep learning curve, especially in obese patients. Whereas hand-assisted laparoscopic surgery (HALS), a hybrid endoscopic technique incorporating elements of both laparoscopic and open techniques, can overcome these existing limitations. Since the first report in 1995 [8], HALS has been demonstrated more efficient than standard laparoscopic surgery as far as operating time and conversion rate were concerned [9–13], with oncological clearance comparable to open colorectal resection [14, 15]. However, to date, little solid evidence exists in support of HALS for mid and low rectal cancer in terms of locoregional recurrence and long-term survival outcomes. Thus, a comparison of HALS versus the open approach for mid and low rectal cancer was performed with long-term follow-up.

## Methods

Between February 2005 and October 2010, consenting patients who underwent HALS for the treatment of mid and low rectal adenocarcinoma (≤12 cm from the anal verge) were prospectively registered. The HALS group was matched against a contemporary open resection series with mid and low rectal adenocarcinoma (≤12 cm from the anal verge) that declined to undergo HALS or LAP at the ratio 1:1 by clinical stage, tumor location, neoadjuvant therapy, gender, age, and body mass index (BMI). Patients with tumor infiltration to the adjacent organs or structures (T4), those with multiple primary colorectal carcinomas, those with familial adenomatous polyposis, those with concurrent distant metastases, those with other malignant diseases, those with recurrent rectal cancer, or those with intestinal obstruction were excluded. This study was approved by the ethics committee of the First Affiliated Hospital, College of Medicine, Zhejiang University.

All patients underwent a physical examination, total colonoscopy plus biopsy. The anesthetist assessed all patients before the operation and assigned an American Society of Anesthesiologists (ASA) grade. Thoracic and abdominal computed tomography (CT) and pelvic magnetic imaging or transrectal ultrasound were performed for preoperative clinical staging. All patients received the same postoperative treatment protocols, including pain control, nutrition support, postoperative rehydration, early ambulation, and early feeding. The discharge criteria included self-feeding, free ambulation, and only mild pain in the wounds.

Patients with low (0–5 cm) rectal cancers were suggested to receive neoadjuvant chemoradiation if the preoperative TNM stage was stage II or III. Other rectal cancers (5.1–12 cm) were considered selectively based on the extent of the disease. The regimen of neoadjuvant chemoradiotherapy was as follows: 4500 cGy in 25 fractions to the pelvis in 5 weeks and a 540 cGy boost in three fractions to the primary tumor, with oral capecitabine concurrently at a dose of 1000–1500 mg/m^2^ per day. The operation was carried out 6–8 weeks after the completion of neoadjuvant treatment by the same surgical team. Postoperative patients with stage III disease received adjuvant chemotherapy.

### Surgical techniques

Seven surgeons (JJL, JHX, WBC, XMX, FLL, XLZ, CZL) performed the operations in the department of Colorectal Surgery at the First Affiliated Hospital of Zhejiang University in the study. Three surgeons (XMX, JJL, WBC) performed the HALS procedures in the study. All three surgeons have extensive laparoscopic experience (>50 hand-assisted laparoscopic colorectal dissections for benign or malignant diseases).

All patients underwent mechanical bowel preparation. All patients underwent total mesorectal excision (TME) with preservation of the hypogastric nerves. For anterior resection, stapled colorectal or handsewn coloanal anastomoses were constructed. An ileostomy was fashioned at the surgeon’s discretion, mainly in patients who had undergone neoadjuvant treatment. The loop ileostomy was reversed with stapled anastomosis technique or hand-suture technique when the stapled anastomosis was not feasible 3 months after the curative surgery. An abdominoperineal excision (APE) was performed when it was not possible to obtain a distal margin of more than 1 cm. Some patients aged >75 years with comorbidities (diabetes, metabolic disorders, anemia) received Hartmann procedure.

Patients who underwent HALS were placed in the Lloyd-Davis position with forced Trendelenburg (30°). The surgeon, utilizing the left hand for retraction and dissection, stood to the patient’s right, whereas the first assistant between the patient’s legs. The monitor was placed on the left side of the patient. HALS was started with a small straight abdominal incision in the midline around the umbilicus to place the hand port (Lap-Disc; Ethicon, Guaynabo, Puerto Rico), which was used for the removal of the resected specimen. A pneumoperitoneum was created with a pressure of 12–15 mmHg, and two additional trocars were placed: a 10-mm trocar in the suprapubic region for the laparoscope and a 12-mm working port in the lower right quadrant. The using of a third 5-mm trocar in the left was at the surgeon’s discretion. Ligation of lymphovascular pedicles and mobilization of the colon and/or rectum was performed intracorporeally along the “Holy plane” down to the pelvic floor. The hypogastric nerves, the pelvic parasympathetic plexus, and the ureters were carefully safeguarded. The lower rectum was transected with endoscopic linear stapler introduced through the right lower quadrant port. Intracorporeal double-stapled colorectal anastomosis technique or hand-suture coloanal anatomosis were performed tans-anally. The splenic flexure was mobilized if extra bowel length was needed to facilitate the construction of a tension-free anastomosis.

The open group followed the same oncologic principles as the HALS procedures with a median incision from the symphysis pubis to the navel. Intestinal separation was conducted according to TME principle as in the HALS group.

All specimens were analyzed by experienced pathologists, who assessed harvested lymph nodes, the distal margin involvement (tumor reaching the distal section), and the circumferential margin involvement (a distance of 1 mm or less from the tumor to the mesorectal fascia).

The sex, age, height, weight, ASA grade, tumor location, and preoperative TNM stage of the patients were recorded, as were operating time, incision length, blood loss, postoperative bowel function, complications, analgesic requirements, length of postoperative stay, and pathological information. Short- and long-term complications were recorded using either medical records or by follow-up.

Symptoms of anastomotic leakage were evaluated by digital examination (DE) and proctoscopy followed by abdominal CT scan. Clinical anastomotic leakage was determined by peritonitis, pelvic abscess, abdominal drain discharge of feces or pus, discharge of pus from the rectum, or rectovaginal fistula. CT-enema was performed for suspected patients postoperatively if the anastomotic dehiscence was not detectable by DE.

Locoregional recurrence was defined as reappearance of a tumor in the surgical pelvic field. Locoregional recurrence was confirmed by histological examination. Distant metastases were diagnosed by radiological and/or histological examination. Further treatments of resection or percutaneous radiofrequency ablation were at the multidisciplinary team’s discretion.

### Follow-up

Patients were followed up as outpatients every 3 months within the first 2 years, every 6 months for the next 3 years subsequently, and at 6 months or yearly thereafter. At each visit, they received a physical examination and general blood tests. Every 6 months, they alternated between thoracic and abdominal CT or abdominal ultrasonography and chest radiography. A complete colonoscopy was performed at the 1-year visit.

### Statistical analyses

All data were analyzed by the software package GraphPad Prism 6.02 (GraphPad Software, San Diego, CA, USA) on an intention-to-treat basis. Pearson’s *χ*^2^-test (or Fisher’s exact test when appropriate) and Mann–Whitney *U* test were performed to compare categorical and numerical data, respectively. Each test was two-tailed, and *P* values < 0.05 were considered to be significant. The data are expressed as the means ± standard deviations or numbers with percentages in parentheses unless otherwise indicated.

Survival rates were calculated with the Kaplan–Meier estimation method. Survival curves were compared with the log rank test. For the calculation of disease-free survival (DFS), patients who died without disease recurrence or metastases were censored at the time of death.

## Results

One hundred and sixteen patients who underwent HALS were compared with 116 patients who underwent open rectal resection. The two groups had comparable demographic data (Table 1). The operative results are summarized in Table 2. The rate of sphincter-preserving surgery was similar in the two groups (*P* = 0.502). Defunctioning ileostomy was created in 54.9 % of patients who underwent sphincter-preserving surgery, again with no differences between groups (*P* = 0.471). The morbidity after the closure of loop ileostomy was comparable between groups. Blood loss and wound infection were significantly less in HALS group (*P* < 0.001 and P = 0.015, respectively). Mean operating time was 34 min longer for HALS than open surgery (*P* < 0.001). Return to oral diet and postoperative hospital stay were longer in the open group (*P* < 0.001). The rates of anastomotic dehiscence, which occurred in eight (8.9 %) patients in the open group and seven (8.2 %) in HALS group, did not differ significantly (*P* = 0.877). There was one death (0.9 %) in the open group due to respiratory infection and multiple organ failure. One death in the HALS group was due to anastomotic dehiscence and subsequent septic shock. There was a similar incidence between the open and HALS groups with respect to chest infection, urinary retention, rectovaginal fistula, urinary fistula, incision hernia, and parastomal hernia. One operation (0.9 %) in the HALS group was converted to an open procedure. The patient with distal margin involved received a salvage surgery of APE.

**Table 1:** Patient characteristics

	Open	HALS	*P* value
	(*n* = 116)	(*n* = 116)	
Sex ratio (M/F)	71/45	68/48	0.688^a^
Age (years)	64 ± 11	61 ± 16	0.310^a^
Body mass index (kg/m^2^)	24.1 ± 5.0	23.3 ± 3.1	0.315^a^
ASA grade			0.652
I	42 (36.2)	39 (33.6)	
II	36 (31.0)	45 (38.8)	
III	33 (28.5)	28 (24.1)	
IV	5 (4.3)	4 (3.5)	
Tumor distance from AV (cm)			0.311^a^
0–5 cm	37 (31.9)	30 (25.9)	
5.1–12 cm	79 (68.1)	86 (74.1)	
Preoperative staging			0.861^a^
I	32 (24.1)	30 (25.9)	
II	41 (31.9)	45 (38.8)	
III	43 (33.6)	41 (35.3)	
Neoadjuvant CRT	33(28.4)	28 (24.1)	0.456

*ASA* American Society of Anesthesiologists, *AV* Anal verge, *CRT* chemoradiotherapy

^a^Matched parameters

**Table 2:** Operative data and postoperative complications

	Open	HALS	*P* value
	(*n* = 116)	(*n* = 116)	
Surgical procedure			0.502
Dixon	90 (77.6)	85 (73.2)	
Hartmann	12 (10.3)	18 (15.5)	
APE	14(12.1)	13 (11.2)	
Anastomosis^b^			0.758
Stapled	82 (90.2)	80 (94.1)	
Handsewn	8 (9.8)	5 (5.9)	
Loop ileostomy^b^	47 (52.2)	49 (57.6)	0.471
Morbidity of loop ileostomy closure	10 (21.3)	9 (18.4)	0.721
Incision length (cm)	16 ± 2	6 ± 1	<0.001
Operative time (minutes)	126 ± 21	160 ± 36	<0.001
Blood loss (ml)	392 ± 95	262 ± 136	<0.001
Duration of narcotic analgesia(days)	3.2 ± 1.2	2.0 ± 1.1	<0.001
Return to oral diet (days)	4.3 ± 1.6	2.9 ± 1.5	<0.001
Length of postoperative stay (days)	8.5 ± 2.5	6.5 ± 2.0	<0.001
Complications	36 (31.0)	31(26.7)	0.469
Anastomotic leakage^b^	8 (8.9)	7 (8.2)	0.877
Anastomotic bleeding^b^	5 (5.6)	3 (3.5)	0.721^a^
Rectovaginal fistula^b^	2 (2.2)	2 (2.4)	1.000^a^
Wound infection^c^	15 (12.9)	4 (3.4)	0 · 015^a^
Chest infection^c^	7 (6.0)	3 (2.6)	0 · 333^a^
Urinary fistula^c^	0 (0)	2 (1.7)	0.498^a^
Urinary retention^c^	9 (7.8)	6 (5.2)	0.423
Intestinal obstruction^c^	6 (5.2)	11 (9.5)	0.208
Incision hernia^c^	4 (3.4)	1 (0.9)	0 · 370^a^
Parastomal hernia^c^	2 (1.7)	2 (1.7)	1.000^a^
Postoperative death^c^	1 (0.9)	1 (0.9)	1.000^a^
Conversion to open surgery		1	

*APE* Abdominoperineal excision

^a^Fisher Exact test

^b^Calculated from the operation of Dixon

^c^Calculated from the curative surgery

Pathological examination data of the specimen were listed in Table 3. Involvement of the circumferential margin and distal margin and the number of isolated lymph nodes were not significantly different between the 2 groups. In the open group for patients with CRM involved, five had locoregional recurrences and three developed distal metastases. While in the HALS group, the four patients with CRM involved developed both locoregional and distal recurrences.

**Table 3:** Pathological characteristics after curative resection

	Open	HALS	*P* value
	(*n* = 116)	(*n* = 116)	
Pathological TNM stage			0.711^b^
CR	1 (0.9)	1 (0.9)	
0	2 (1.7)	1 (0.9)	
I	30 (25.9)	27 (23.3)	
II	46 (39.6)	52 (44.8)	
III	37 (31.9)	35 (30.2)	
T stage			0.641^c^
T0	1 (0.9)	1 (0.9)	
Tis	2 (1.7)	1 (0.9)	
T1	11 (9.5)	17 (14.6)	
T2	31 (26.7)	29 (25)	
T3	71 (61.2)	68 (58.6)	
N stage			0.578
N0	79 (68.1)	81 (69.8)	
N1	17 (14.7)	12 (10.4)	
N2	20 (13.8)	23 (12.9)	
Grade of differentiation			0.687
Well	12 (10.3)	18 (15.5)	
Moderate	80 (69.0)	75 (64.7)	
Poor	17 (1.7)	16 (13.8)	
Mucinous	5 (4.3)	6 (5.2)	
Unknown	2	1	
Isolated lymph nodes	12.2 ± 3.5	13.5 ± 4.6	0.342
CRM			0.374^a^
Involved (≤1 mm)	8 (5.4)	4 (3.4)	
Noninvolved (>1 mm)	99 (85.3)	101 (87.1)	
Missing	9	11	
Distal margin involved^c^	0 (0)	1 (1.0)	1.000^a^

*CR* Complete response, *CRM* Circumferential resection margin

^a^Fisher exact test

^b^CR, TNM stage 0, and stage I were combined for statistical analysis

^c^T0, Tis, and T1 were combined for statistical analysis

**Fig. 1:**
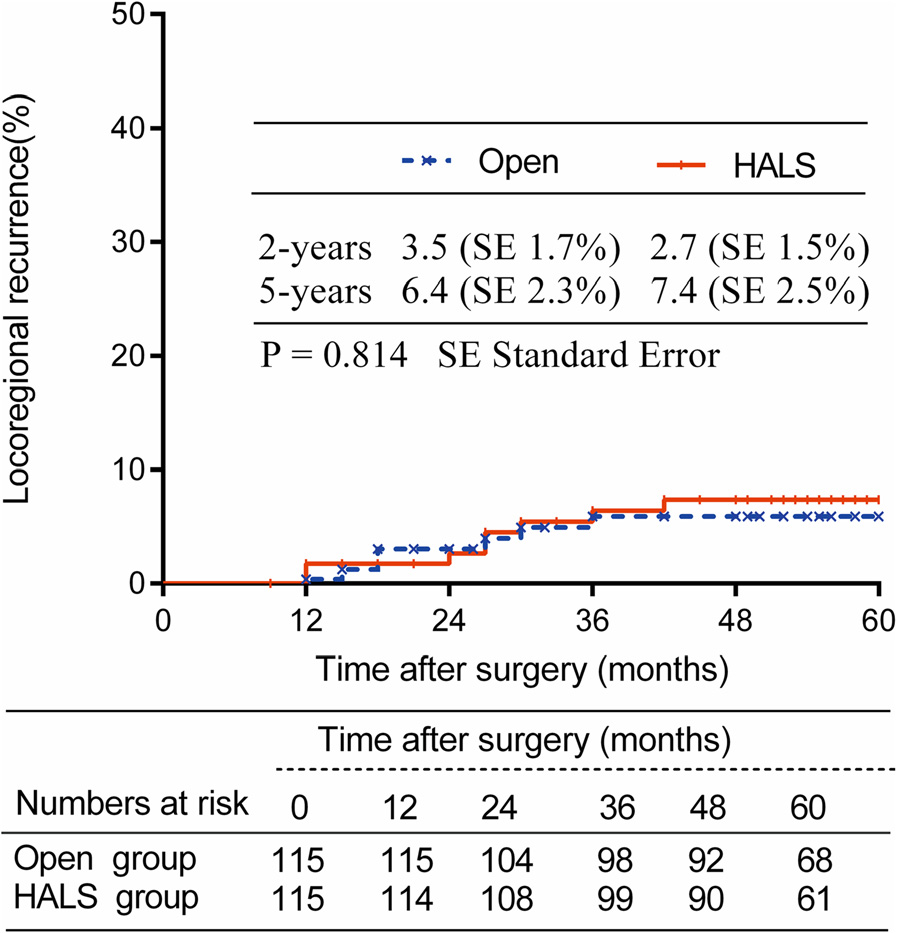
Locoregional recurrence in Open and HALS groups

**Fig. 2:**
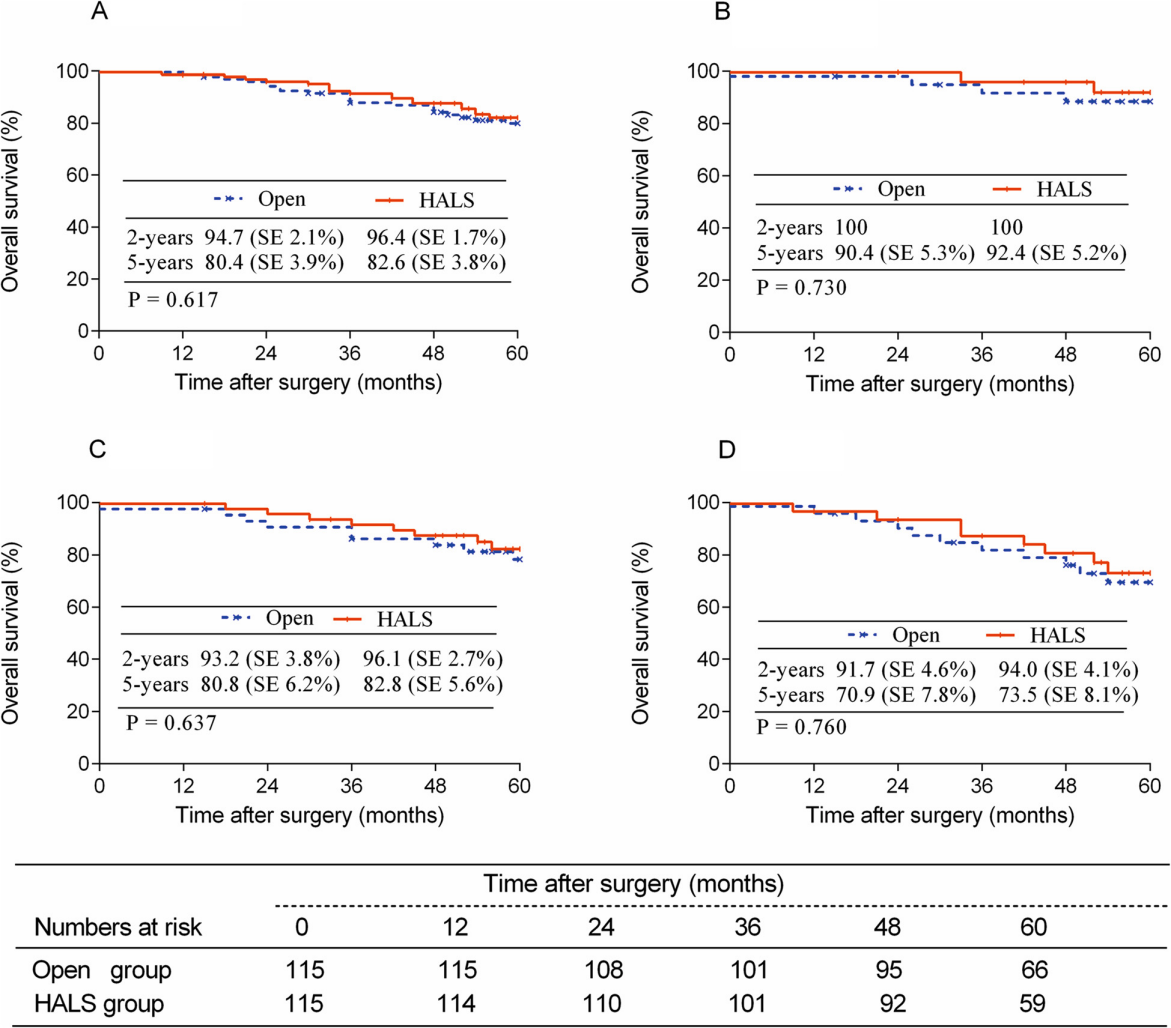
Overall survival rate of patients with HALS or Open surgery ((**a**) all stages; (**b**) stage 0-I; (**c**) stage II; (**d**) stage III)

**Fig. 3:**
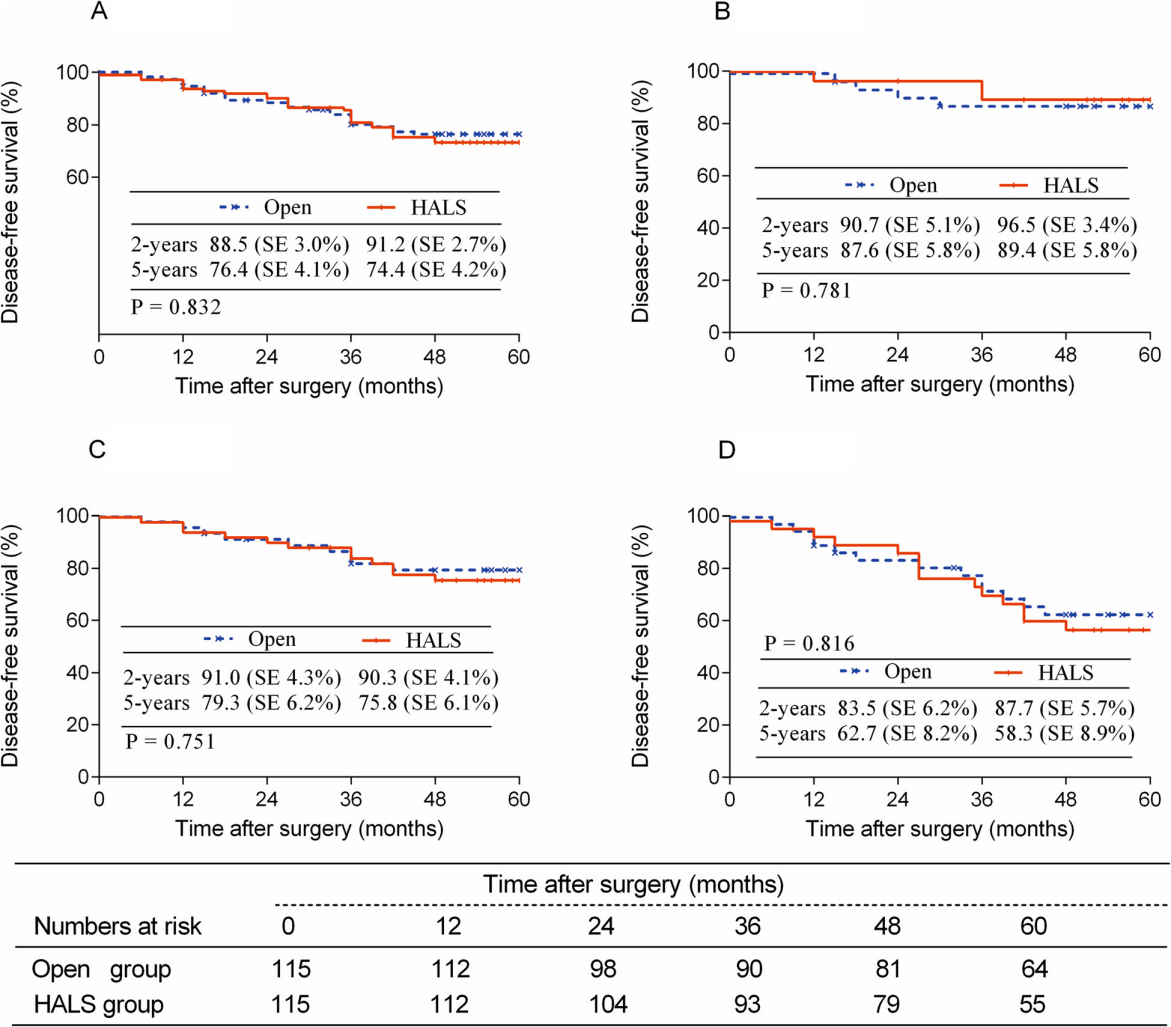
Disease-free survival rate of patients with HALS or Open surgery ((**a**) all stages; (**b**) stage 0-I; (**c**) stage II; (**d**) stage III)

The duration of follow-up was 63.3 ± 23.4 months for the open group and 61.5 ± 21.9 months for the HALS group (*P* = 0.516). Locoregional recurrence was similar between groups (*P* = 0.814) (Fig. 1). There were no incision or port sites recurrences. Nineteen patients in the open group and twenty-two patients in the HALS group developed distal metastases. The overall 5-year survival rate was 80.4 % in the open group and 82.6 % in the HALS group (*P* = 0.617) (Fig. 2a). The subgroup survival analysis according to the stage was similar between both groups (Fig. 2b–d). The 5-year DFS rate was 76.4 % in the open group and 74.4 % in the HALS group (*P* = 0.832) (Fig. 3a), with a subgroup analysis similar, too (Fig. 3b–d).

## Discussion

This study suggested that HALS was associated with less blood loss, faster gastrointestinal recovery, and shorter postoperative hospital stay, without compromise of oncological clearance compared with open laparotomy for mid and low rectal cancer. Notably, the rates of locoregional recurrence, DFS and OS were similar between the two groups through long-term follow-up.

The potential advantages of HALS have been delineated previously in colorectal surgery [14, 16–20]. The HALS Study Group concluded that HALS retained the benefits of minimally invasive surgery and may allow the surgeon to carry out complicated procedures efficiently [18]. Surgeons can easily locate tumors by tactile feedback and decrease iatrogenic injuries. Moreover, the surgeon’s left hand inside the abdomen greatly facilitates the placement of the linear stapler and the mobilization of the left colon [21]. Not surprisingly, as a consequence, HALS group resulted in shorter operating time and comparable lengths of hospitalization stay compared with LAP although the incision of HALS was a little bit longer [12]. Furthermore HALS resulted in low conversion rate being varied from 0 to 10 % [15], which was consistent with our data. nevertheless, in published LAP series, the conversion rate to open surgery varied from 1.2 to 29 % (1.2 % in the COREAN trial [4], 7.5 % in Ng’s study [2], 7.9 % in Lujan’s study [3], 17 % in the COLOR II trial [22], and 29 % in the CLASICC trial [23]). What is particularly worth mentioning is that the conversion rate of LAP increased with increasing BMI [24]. For obese patients (BMI > 30) the conversion rate could reach as high as 31.9 % [24]. Whereas, in a study carried out in Cleveland Clinic in obese patients (BMI > 30), HALS resulted in lower conversion rate compared with LAP (3.5 vs. 12.7 %). Therefore, Heneghan et al. suggested HALS as a first-line approach for colorectal surgery in obese patients [9]. It also indicated that conversion from LAP to HALS in difficult or complicated cases should be considered to reduce conversion rate to open surgery.

However, HALS port placement and trocar arrangement varies with different surgeons [16–20]. Some authors [17] preferred to place the Gelport in the low midline or Pfannenstiel incision. Our experience showed that midline HALS port placement around the umbilicus greatly facilitated four-quadrant dissection, especially when the splenic flexure mobilization was required. The surgeon’s hand in the abdomen greatly facilitates retraction, dissection, and hemostasis. If inappropriate, however, the hand might be cumbersome rather than helpful. In addition to appropriate positioning of the HALS port, correct trocar placement is of vital importance. The principles of instrument triangulation should be adhered to in trocar arrangements. The laparoscope port site may be better when placed in the midline in the suprapubic region allowing views of the entire pelvic area and the whole rectum by adjusting the 30° lens of the laparoscope, for otherwise, the hand is unable to be helpful and can become a hindrance. Under this protocol, the left side trocar was rarely needed in addition to the working port at the right side.

It is worthy to be noted that HALS surgery cannot decrease the anastomotic leakage rate. Defunctioning ileostomy was required to mitigate the serious sequelae of an anastomotic leak. However, the morbidity after loop ileostomy closure remains high. More recently, tube ileostomy has been performed to avoid the construction of loop ileostomy, and the preliminary results were promising [25–27].

Previous studies and meta-analyses have shown that laparoscopic TME was an oncologically correct technique. The rate of distal and circumferential margin involvement and the number of isolated lymph nodes were reported similar for both laparoscopic and open techniques [28–30]. Nevertheless, data on the number of harvested lymph nodes, positive margin rate, and recurrence rate were seldom reported in HALS study. The pathological data in our study suggested that HALS was oncologically acceptable in the mid and low rectal cancer surgery.

Despite the short-term benefits, the long-term survival outcome of HALS for mid and low rectal cancer was rarely reported, which, however, is mandatory for establishing the value of HALS in the surgical treatment. The short-term benefits of HALS should not be compromised by the incidence of locoregional recurrence and survival. There were discrepancies between LAP and open surgery group for rectal cancer in terms of survival outcomes. In the CLASICC trial, the 5-year OS rates for the rectal cancer patients were not statistically different between the LAP (60.3 %) and open (52.9 %) groups [1]. In the COREAN trial, LAP group provides similar outcomes of three-year DFS as open surgery for locally advanced rectal cancer after preoperative chemoradiotherapy [4]. Lujan et al. [3] reported similar results. These results were in line with our findings. Ng et al. [2] reported that the LAP group had a survival advantage over the open approach. However, its small sample size did not allow convincing conclusion to be drawn.

We must note, however, that HALS technique has limitations. First of all, HALS requires additional use of hand port, which increases the surgical cost and may result in fatigue and numbness of the surgeon’s hand in some cases, though some reported that the overall cost was equivalent [31]. Additionally, HALS resulted in longer operative duration, which may be partly owing to the complexity of rectal surgery. The increased handling and mobilization of the bowel was speculated to result in the development of postoperative ileus and intra-abdominal adhesions, whereas in our study, the increased duration is unlikely to cause negative impacts on patients clinically. Moreover, the study was limited by the absence of data concerning genitourinary functional outcomes and non-randomization design. Future well-designed multicenter studies with more patients are needed to allow a more convincing evaluation, and further comparative study to evaluate HALS vs. LAP for mid and low rectal cancer, especially in obese patients, is also required.

## Conclusions

In conclusion, surgeons now have many different options to achieve a minimally invasive operation for colorectal resection, and our data suggests that HALS for mid and low rectal cancer can improve postoperative recovery, reduces blood loss and postoperative hospital stay, and provides similar long-term survival outcomes compared with open laparotomy.
